# Performance of ChatGPT in Ophthalmic Registration and Clinical Diagnosis: Cross-Sectional Study

**DOI:** 10.2196/60226

**Published:** 2024-11-14

**Authors:** Shuai Ming, Xi Yao, Xiaohong Guo, Qingge Guo, Kunpeng Xie, Dandan Chen, Bo Lei

**Affiliations:** 1 Department of Ophthalmology Henan Eye Institute, Henan Eye Hospital Henan Provincial People's Hospital Zhengzhou China; 2 Eye Institute Henan Academy of Innovations in Medical Science Zhengzhou China; 3 Henan Clinical Research Center for Ocular Diseases People’s Hospital of Zhengzhou University Zhengzhou China; 4 Department of Ophthalmology The First Affiliated Hospital of Zhengzhou University Zhengzhou China

**Keywords:** artificial intelligence, chatbot, ChatGPT, ophthalmic registration, clinical diagnosis, AI, cross-sectional study, eye disease, eye disorder, ophthalmology, health care, outpatient registration, clinical, decision-making, generative AI, vision impairment

## Abstract

**Background:**

Artificial intelligence (AI) chatbots such as ChatGPT are expected to impact vision health care significantly. Their potential to optimize the consultation process and diagnostic capabilities across range of ophthalmic subspecialties have yet to be fully explored.

**Objective:**

This study aims to investigate the performance of AI chatbots in recommending ophthalmic outpatient registration and diagnosing eye diseases within clinical case profiles.

**Methods:**

This cross-sectional study used clinical cases from *Chinese Standardized Resident Training–Ophthalmology (2nd Edition)*. For each case, 2 profiles were created: patient with history (Hx) and patient with history and examination (Hx+Ex). These profiles served as independent queries for GPT-3.5 and GPT-4.0 (accessed from March 5 to 18, 2024). Similarly, 3 ophthalmic residents were posed the same profiles in a questionnaire format. The accuracy of recommending ophthalmic subspecialty registration was primarily evaluated using Hx profiles. The accuracy of the top-ranked diagnosis and the accuracy of the diagnosis within the top 3 suggestions (do-not-miss diagnosis) were assessed using Hx+Ex profiles. The gold standard for judgment was the published, official diagnosis. Characteristics of incorrect diagnoses by ChatGPT were also analyzed.

**Results:**

A total of 208 clinical profiles from 12 ophthalmic subspecialties were analyzed (104 Hx and 104 Hx+Ex profiles). For Hx profiles, GPT-3.5, GPT-4.0, and residents showed comparable accuracy in registration suggestions (66/104, 63.5%; 81/104, 77.9%; and 72/104, 69.2%, respectively; *P*=.07), with *ocular trauma*, *retinal diseases*, and *strabismus and amblyopia* achieving the top 3 accuracies. For Hx+Ex profiles, both GPT-4.0 and residents demonstrated higher diagnostic accuracy than GPT-3.5 (62/104, 59.6% and 63/104, 60.6% vs 41/104, 39.4%; *P*=.003 and *P*=.001, respectively). Accuracy for do-not-miss diagnoses also improved (79/104, 76% and 68/104, 65.4% vs 51/104, 49%; *P*<.001 and *P*=.02, respectively). The highest diagnostic accuracies were observed in *glaucoma*; *lens diseases*; and *eyelid, lacrimal, and orbital diseases*. GPT-4.0 recorded fewer incorrect top-3 diagnoses (25/42, 60% vs 53/63, 84%; *P*=.005) and more partially correct diagnoses (21/42, 50% vs 7/63 11%; *P*<.001) than GPT-3.5, while GPT-3.5 had more completely incorrect (27/63, 43% vs 7/42, 17%; *P*=.005) and less precise diagnoses (22/63, 35% vs 5/42, 12%; *P*=.009).

**Conclusions:**

GPT-3.5 and GPT-4.0 showed intermediate performance in recommending ophthalmic subspecialties for registration. While GPT-3.5 underperformed, GPT-4.0 approached and numerically surpassed residents in differential diagnosis. AI chatbots show promise in facilitating ophthalmic patient registration. However, their integration into diagnostic decision-making requires more validation.

## Introduction

Artificial intelligence (AI) has significantly advanced in health care, particularly in many areas of ophthalmology [1,2]. ChatGPT (OpenAI) [3] is a generative AI featuring a chatbot interface. Benefiting from its expansive knowledge base and complex parameterization, it enables users to input queries and receive responses that showcase advanced, humanlike logic. Since its launch in November 2022, ChatGPT has quickly amassed a substantial user base. It was recognized as having the potential to revolutionize not only ophthalmology but also the entire medical field in diverse aspects [4], including patient care, health care professionals and systems, research, and education and training [5]. Its performance was highlighted in patient triage proficiency [6], scientific writing [7], operative notes writing [8], and passing the ophthalmology specialist licensing examination [9].

Specialized eye hospitals in China, especially tertiary ones, frequently face patient overcapacity. With limited knowledge of eye health, patients could encounter difficulties in choosing the right subspecialty department when registering. User-friendly chatbot such as ChatGPT could provide registration suggestions based on the patients’ chief complaints and medical histories and, thus, significantly ease these challenges and reduce health care resource wastage due to unsuitable registrations. However, the role of ChatGPT in classifying diseases into ophthalmic subspecialties and thus facilitating patient registration remains unexplored.

ChatGPT has exhibited encouraging results in diagnosing eye diseases within specific subspecialties, such as corneal and retinal vascular diseases [10,11]. The data for these assessments were sourced from public question banks and case report databases. In diagnosing a diverse range of ophthalmic conditions, ChatGPT failed to match the diagnostic accuracy of ophthalmologists but demonstrated the benefit of a shorter diagnostic time [12]. Further validation studies, particularly in testing ChatGPT’s diagnostic effectiveness for a comprehensive range of eye diseases within the context of clinical practice in China, are essential.

Drawing on typical clinical cases from Chinese Standardized Resident Training (SRT) materials, this study aims to evaluate ChatGPT’s capacity for classifying ophthalmic subspecialties and its diagnostic potential within the Chinese context. Our research seeks to provide insights into whether ChatGPT can effectively assist patients with appropriate registrations and support ophthalmologists in clinical decision-making.

## Methods

### Ethical Considerations

The Institutional Review Board of Henan Provincial People’s Hospital determined that this in silicon research did not involve direct interaction with real-world human subjects, nor did it require the collection of new human data. Accordingly, an ethics exemption was granted for this study. The case information used was derived from publicly available sources and published materials. At the time of data collection, GPT-4.0 was publicly available by paid subscription through ChatGPT Plus.

### Data Source

The clinical cases for our study were sourced from *Chinese Standardized Resident Training–Ophthalmology (2nd Edition)*, which is an official resource conforming to SRT content and standards, as well as the theoretical assessment guidelines of the National Health Commission of China. Unlike traditional undergraduate textbooks, SRT materials are specifically designed to focus on problem-based learning and case-based learning. They feature a variety of typical real-world cases from various ophthalmic subspecialties, making them particularly suitable for interaction with ChatGPT’s chatbot interface, which is designed to handle complex, real-life queries.

In this study, we gathered 121 cases from 12 ophthalmic subspecialties, creating 2 profiles per case: patient with history (Hx) and patient with history and examination (Hx+Ex) [12]. The “history” portion comprised gender, age, chief complaints, and medical history. When necessary, past medical, familial, and systemic disease details were also added. The “examination” portion covered general ophthalmic assessments such as visual acuity and intraocular pressure, alongside diagnostics such as slit lamp biomicroscopy, a range of ophthalmic imaging (eg, fundus photography, orbital computed tomography [OCT], and fluorescein angiography), and specialized imaging for eye tumors and traumas (OCT and magnetic resonance imaging). The inclusion criteria mandated comprehensive historical and ophthalmology-related chief complaint details, documented examination results, and official case analyses with accurate subspecialty classifications and unique diagnoses.

After excluding 17 cases, a total of 104 cases were retained for further analysis. The reasons for excluding the 17 cases included (1) the lack of medical history or the presence of final diagnostic–like terms in the medical history, which may bias the assessment (6 cases); (2) the lack of textual descriptions of examination results (6 cases); (3) chief complaints not related to ophthalmic symptoms or cases referred from other departments (3 cases); and (4) unclear or non–ophthalmology-related diagnoses (2 cases). The mean age was 36.8 (SD 21.7) years, with male patients comprising 55.8% (58/104) of the cases. Notably, each case had a predominantly unique final diagnosis. The 3 most prevalent diagnoses, classified into subspecialties, were *eyelid, lacrimal, and orbital diseases*; *retinal diseases*; and *strabismus and amblyopia* (Table 1). Given that the original 104 profiles were ordered based on the ophthalmic subspecialty, a new case numbering system was established by randomly assigning descending numerical values between 0 and 1 and arranging them accordingly.

**Table 1:** Classification of subspecialties in the 104 clinical cases.

Ophthalmic subspecialty			Clinical cases (n=104), n (%)
Eyelid, lacrimal, and orbital diseases			18 (17)
Retinal diseases			
	Nonheritable	15 (13)	
	Heritable	6 (6)	
Strabismus and amblyopia			10 (10)
Corneal and ocular surface diseases			9 (9)
Refractive errors			7 (7)
Scleral and uveal diseases			6 (6)
Ocular trauma			6 (6)
Eye tumors			
	Eyelid, lacrimal, and orbital tumors	6 (6)	
	Scleral and uveal tumors	4 94)	
	Retinal tumors	1 (1)	
Glaucoma			5 (5)
Vitreous diseases			3 (4)
Lens diseases			4 (4)
Neuro-ophthalmology			4 (4)
Total			104 (100)

### Testing Process

**Figure 1:**
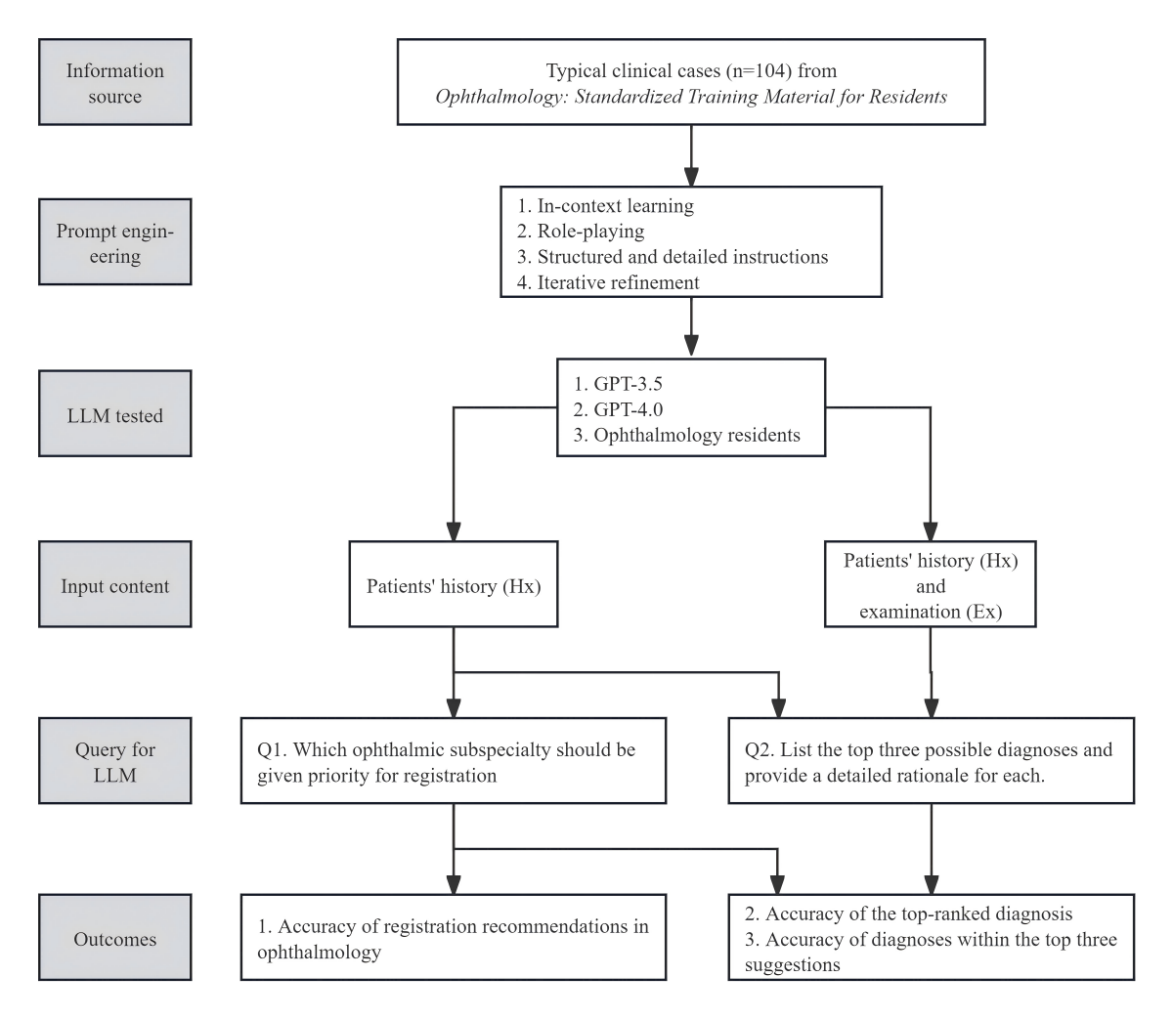
The design and analytical framework of the study. LLM: large language models.

A total of 208 clinical profiles (104 Hx and 104 Hx+Ex profiles) in Chinese were tested from March 5 to 18, 2024. The tested AI chatbots included ChatGPT versions 3.5 and 4.0 (GPT-3.5 and GPT-4.0, respectively), and 3 ophthalmology residents were also tested. Initially, ChatGPT was assigned a system role to emulate a professional ophthalmologist. Each clinical case scenario was entered in Chinese independently, followed by 2 questions: “Q1. Which ophthalmic subspecialty should be given priority for registration?” and “Q2: List the top three possible diagnoses and provide a detailed rationale for each.” Q1 aimed to elicit the chatbot’s triage recommendations for subspecialty registration, while Q2 focused on extracting the chatbot’s leading and differential diagnosis proposals. For the Hx profiles, both questions were asked, and the AI was informed of the available subspecialties for reference in the prompt. For the Hx+Ex profiles, given that the examination primarily serves for diagnosis, only Q2 was posed. Response history was reset prior to each new query (Figure 1). The detailed prompts and engineering techniques are shown in Multimedia Appendix 1. Based on the structure provided by these prompts, we found that ChatGPT’s responses generally adhered to the fixed template specified in our prompts. Consequently, we opted to input each case continuously in a single chat session. However, for the Hx and Hx+Ex profiles, as well as for tests conducted with both GPT-3.5 and GPT-4.0, each series of tests was initiated in a new chat session.

For the residents’ test, 2 sets of questionnaires were created following the Hx + Q1 and Hx+Ex + Q2 format. The residents’ evaluations were independent, which were ensured by implementing a blinded assessment process where the residents did not have information about the performance or responses of the AI systems or each other.

### Outcomes and Definition

The study focused on 3 outcomes: accuracy of recommendation for ophthalmic subspecialty registration, accuracy of the top-ranked diagnosis, and accuracy of the diagnosis within the top 3 suggestions (do-not-miss diagnosis). As residents rarely provided 3 possible diagnoses like ChatGPT, they were not evaluated on the accuracy of the do-not-miss diagnosis. The gold standard for judgment was the official diagnosis from *Chinese Standardized Resident Training*–*Ophthalmology (2nd Edition).*

ChatGPT and residents were evaluated based on the same outcome criteria. For ophthalmic subspecialty registration, overlaps exist in some subspecialties, such as *eye tumors* and *retinal diseases*. Recommendations to either category were considered correct. Precision was crucial for diagnosis suggestions corresponding to Hx+Ex profiles. For instance, if the final diagnosis is sympathetic ophthalmia, responses such as panuveitis or herpetic keratitis were marked as incorrect. Similarly, for acute idiopathic optic neuritis or orbital neurilemmoma, responses of optic neuritis or orbital tumor were also considered incorrect. However, for Hx profiles, an exact diagnosis was not required. All responses in the examples provided were considered correct.

Regarding the residents’ performance, a diagnosis was considered correct only if at least 2 out of 3 residents provided the correct diagnosis. This approach emphasizes the importance of consensus in clinical decision-making and reflects the collaborative nature of medical diagnosis in real-world settings.

### Statistical Analysis

Data collection and management were performed using Microsoft Excel software. Statistical analyses were mainly conducted in SPSS (version 26.0.0; IBM Corp). To compare the accuracy of triage and diagnosis across different testing strategies, the Pearson chi-square test or Fisher exact test was applied, depending on the expected frequency counts in the contingency tables. Due to limitations in SPSS for conducting the Fisher exact test on the differences in proportions among 3 groups, we switched to R software (version 4.4.1; R Foundation for Statistical Computing) for this analysis. For post hoc analysis, *P* values were adjusted using the Bonferroni method in pairwise comparisons. Unless otherwise specified, differences were considered statistically significant at *P*<.05.

## Results

### Accuracy of Recommendation for Subspecialty Registration

**Figure 2:**
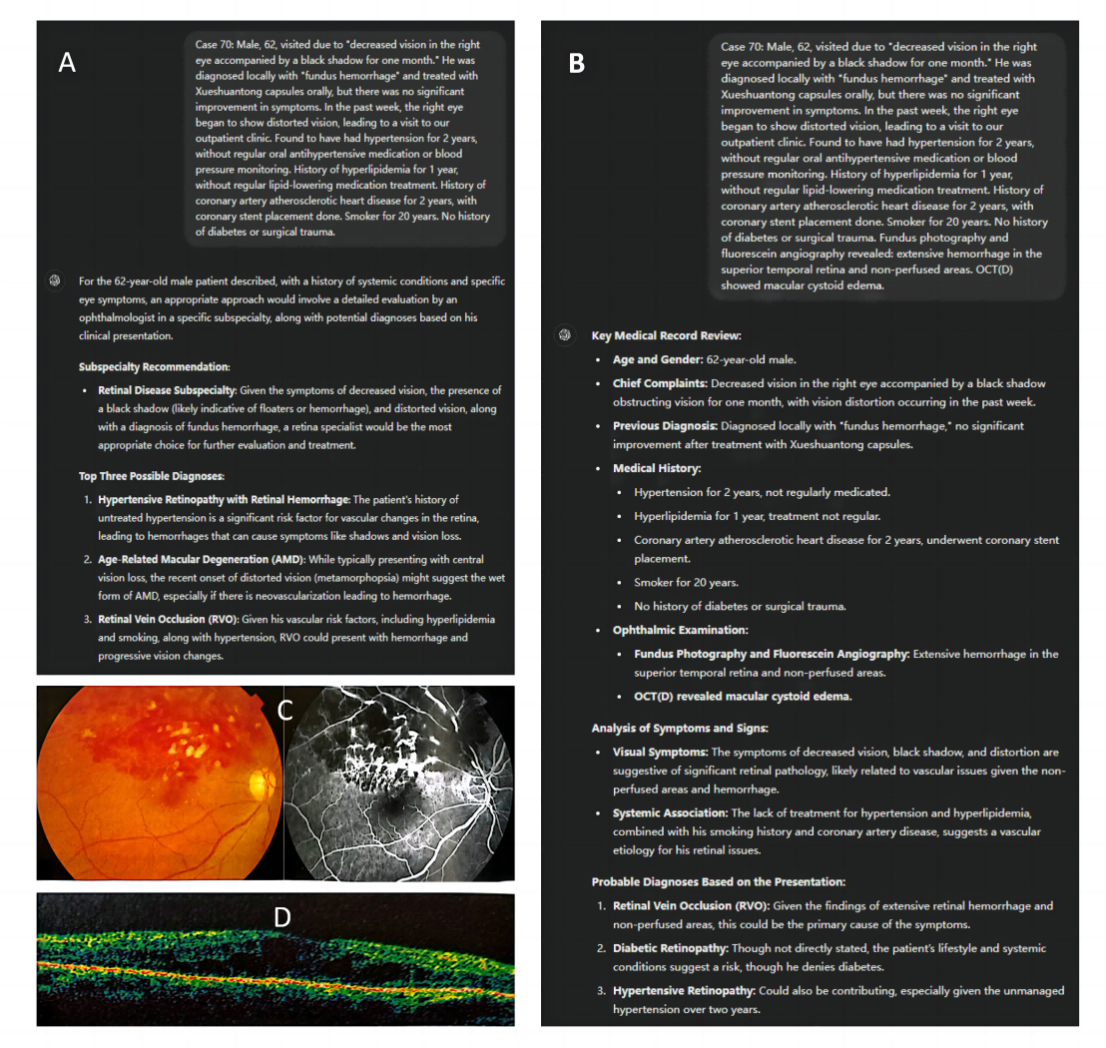
Example profile 70: interaction with and responses of the GPT-4.0 chatbot. (A) When provided with Hx information, GPT-4.0 correctly recommended the ophthalmic subspecialty of “retinal diseases.” (B) When provided with Hx+Ex information, GPT-4.0 gave the top 3 diagnostic suggestions and accurately identified “retinal vein occlusion” as the top-ranked diagnosis. (C) Fundus photography and fluorescein angiography. (D) OCT imaging. (C) and (D) were presented to ChatGPT as textual descriptions of the examination results. Hx: patient with history; Hx+Ex: patient with history and examination; OCT: orbital computed tomography.

For Hx profiles, GPT-3.5, GPT-4.0, and residents demonstrated moderate accuracy in registering patients to the correct ophthalmic subspecialty, with accuracy of 63.5% (66/104), 77.9% (81/104), and 69.2% (72/104), respectively (*P*=.07). Subgroup analysis revealed that *ocular trauma*, *retinal diseases*, and *strabismus and amblyopia* ranked among the top 3 in overall registration accuracy. In contrast, registration accuracy was low for *glaucoma*, *neuro-ophthalmology*, and *lens diseases* (Table 2). The detailed registration recommendations for GPT-3.5, GPT-4.0, and residents are showed in Multimedia Appendix 2. An example of GPT-4.0’s response to subspecialty registration is shown in Figure 2.

**Table 2:** Correct triage recommendations for subspecialty registration for patient history (Hx) profiles.

Ophthalmic subspecialty	GPT-3.5, n (%)	GPT-4.0, n (%)	Residents, n (%)	*P* value
Total (n=104)	66 (63.5)	81 (77.9)	72 (69.2)	.07
Retinal diseases (n=21)	18 (86)	20 (95)	19 (91)	.86
Eyelid, lacrimal, and orbital diseases (n=18)	14 (78)	14 (78)	11 (61)	.44
Eye tumors (n=11)	6 (55)	8 (73)	9 (82)	.52
Strabismus and amblyopia (n=10)	8 (80)	9 (90)	7 (70)	.85
Corneal and ocular surface diseases (n=9)	4 (44)	7 (78)	8 (89)	.10
Refractive errors (n=7)	6 (86)	5 (71)	4 (57)	.83
Ocular trauma (n=6)	6 (100)	6 (100)	6 (100)	—^a^
Scleral and uveal diseases (n=6)	1 (17)	4 (67)	3 (50)	.36
Glaucoma (n=5)	1 (20)	3 (60)	1 (20)	.50
Lens diseases (n=4)	1 (25)	2 (50)	1 (25)	.69
Neuro-ophthalmology (n=4)	1 (25)	1 (25)	1 (25)	>.99
Vitreous diseases (n=3)	0 (0)	2 (67)	2 (67)	.36

^a^Not applicable.

### Accuracy of Diagnosis

For Hx+Ex profiles, both GPT-4.0 and residents demonstrated higher diagnostic accuracy compared to GPT-3.5 (62/104, 59.6% vs 41/104, 39.4%; *P*=.003; and 63/104, 60.6% vs 41/104, 39.4%; *P*=.001, respectively). Similarly, the accuracy of diagnoses within the top 3 suggestions were also higher (79/104, 76% vs 51/104, 49%; *P*<.001; and 68/104, 65.4% vs 51/104, 49%; *P*=.02, respectively). However, there was no statistically significant difference in diagnostic accuracy between GPT-4.0 and residents (79/104, 76% vs 68/104, 65.4%; *P*=.09). Compared to Hx profiles, GPT-4.0 showed improved diagnostic accuracy for Hx+Ex profiles and diagnoses within the top 3 suggestions (62/104, 59.6% vs 42/104, 40.4%; *P*=.007; and 79/104, 76% vs 63/104, 60.6%; *P*=.02, respectively; Table 3). The detailed top-3 predicted diagnoses by GPT-3.5 and GPT-4.0, alongside the composite diagnosis by the 3 residents, are showed in Multimedia Appendix 3. An example of GPT-4.0’s response to diagnosis is shown in Figure 2.

**Figure 3:**
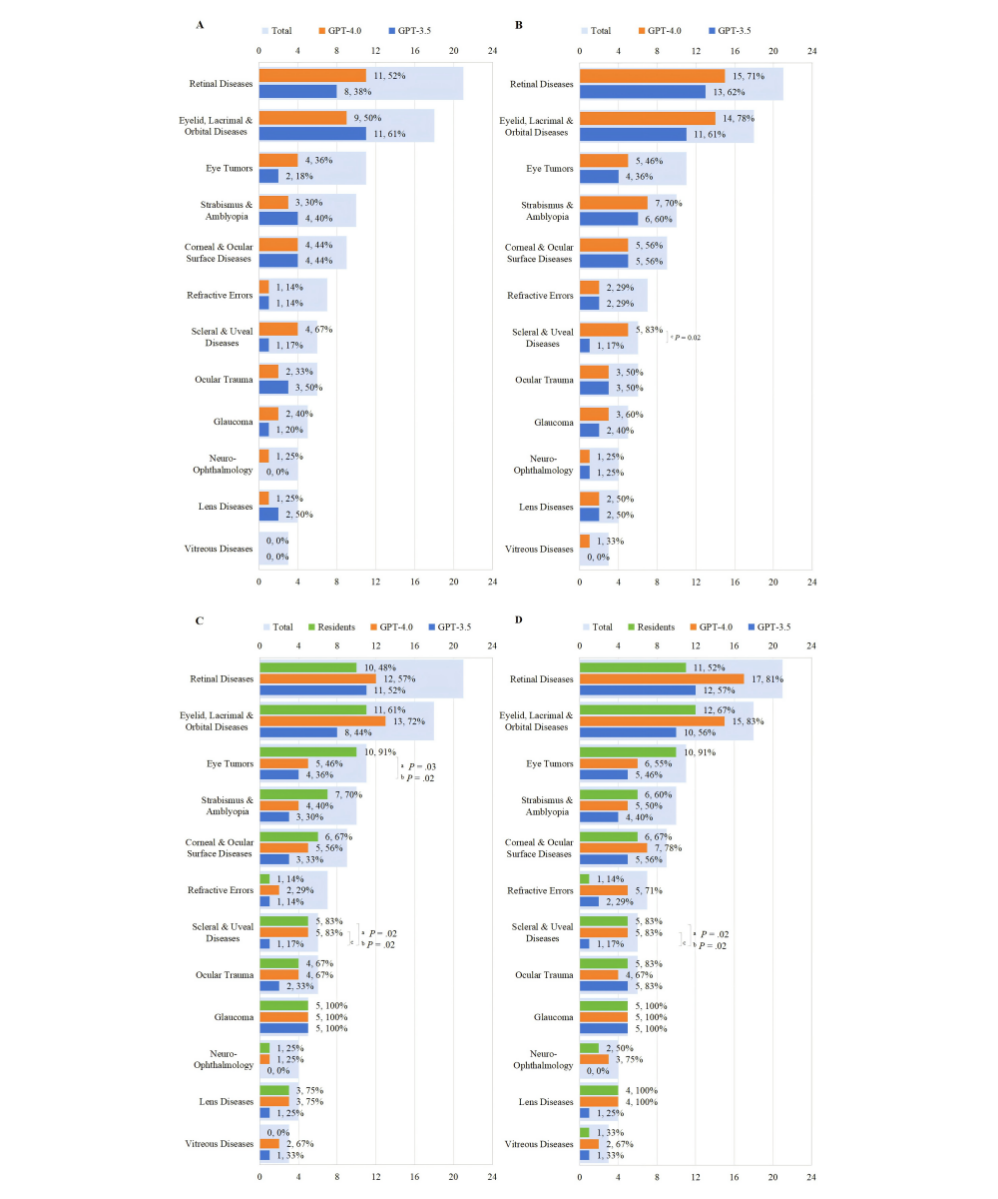
The diagnostic accuracy of GPT-3.5, GPT-4.0, and residents across various ophthalmic subspecialties: (A) accuracy of the top-ranked diagnosis for Hx profiles; (B) accuracy of the diagnosis within the top 3 suggestions for Hx profiles; (C) accuracy of the correct top-ranked diagnosis for Hx+Ex profiles; and (D) accuracy of the diagnosis within the top 3 suggestions for Hx+Ex profiles. “a” indicates significant statistical differences across all 3 groups (GPT-3.5, GPT-4.0, and residents), “b” denotes a significant difference between residents and GPT-3.5, and “c” represents a significant difference between GPT-4.0 and GPT-3.5. Hx: patient with history; Hx+Ex: patient with history and examination.

In the subgroup analysis, both GPT-3.5 and GPT-4.0 exhibited generally lower diagnostic accuracy for Hx profiles. However, for Hx+Ex profiles, there was an overall improvement in diagnosis, particularly for glaucoma. The top 3 subspecialties in overall accuracy were *glaucoma*; *lens diseases*; and *eyelid, lacrimal, and orbital diseases*. In the subspecialties of *eye tumors* and *scleral and uveal diseases*, significant differences were observed in the top-ranked diagnosis accuracy for Hx+Ex profiles among GPT-3.5 , GPT-4.0, and residents (4/11, 36% vs 5/11, 46% vs 10/11, 91%; *P*=.03; and 1/6, 17% vs 5/6, 83% vs 5/6, 83%; *P*=.02, respectively). Notably, for *scleral and uveal diseases*, GPT-3.5 demonstrated a lower accuracy of 17% (1/6) both in the top-ranked diagnosis (*P*=.02) and within the top 3 diagnoses (*P*=.02) compared to GPT-4.0 (5/6, 83%) and the residents (5/6, 83%; Figure 3).

**Table 3:** Secondary outcomes for patient with history (Hx) and patient with history and examination (Hx+Ex) profiles (n=104).

Diagnosis accuracy	GPT-3.5, n (%)	GPT-4.0, n (%)	Residents, n (%)	*P* value
Accuracy A: the top-ranked diagnosis is correct for Hx profiles	37 (35.6)	42 (40.4)^a,b^	—^c^	.48
Accuracy B: the diagnosis is within the top 3 suggestions for Hx profiles	50 (48.1)	63 (60.6)^a^	—	.07
Accuracy C: the top-ranked diagnosis is correct for Hx+Ex profiles	41 (39.4)^d^^,e^	62 (59.6)^b^	63 (60.6)	.002
Accuracy D: the diagnosis is within the top 3 suggestions for Hx+Ex profiles	51 (49)^d,e^	79 (76)	68 (65.4)	<.001

^a^Statistically significant differences for GPT-4.0 when comparing accuracy A vs accuracy C (*P*=.007) and accuracy B vs accuracy D (*P*=.02).

^b^Statistically significant differences for GPT-4.0 when comparing accuracy A vs accuracy B (*P*=.004) and accuracy C vs accuracy D (*P*=.01).

^c^Not applicable.

^d^Statistically significant differences for accuracy C and accuracy D when comparing GPT-3.5 with GPT-4.0 (*P*=.004 and *P*<.001, respectively).

^e^Statistically significant differences for accuracy C and accuracy D when comparing GPT-3.5 with residents (*P*=.002 and *P*=.02, respectively).

### Case Description and Accuracy

For Hx+Ex profiles where the medical history provided past diagnoses related to the final diagnosis, a higher top-ranked diagnosis accuracy was observed in the GPT-4.0 model (7/7, 100% vs 55/97, 57%; *P*=.04). However, case descriptions including past diagnoses unrelated to the final diagnosis and cases requiring ophthalmic examination for a definitive diagnosis did not significantly affect the top-ranked diagnosis accuracy for GPT-3.5, GPT-4.0, and the residents (all *P*>.21; Table 4).

**Table 4:** Case characteristics and their association with the top-ranked diagnosis accuracy for patient with history and examination (Hx+Ex) profiles.

Cases characteristics		GPT-3.5	*P* value	GPT-4.0	*P* value	Residents	*P* value
Prescence of medical history A^a^, n (%)			.43		.04		.70
	No (n=97)	36 (37)		55 (57)		58 (60)	
	Yes (n=7)	4 (57)		7 (100)		5 (71)	
Prescence of medical history B^b^, n (%)			.73		.74		>.99
	No (n=95)	36 (38)		56 (59)		57 (60)	
	Yes (n=9)	4 (44)		6 (67)		6 (67)	
Prescence of characteristics C^c^, n (%)			.21		.29		>.99
	No (n=51)	24 (47.1)		31 (60.8)		31 (61)	
	Partly (n=20)	6 (30)		9 (45)		12 (60)	
	Yes (n=33)	10 (30.3)		22 (66.7)		20 (61)	

^a^Medical history A: descriptions of past diagnoses related to the final diagnosis.

^b^Medical history B: description of past diagnoses unrelated to the final diagnosis.

^c^Characteristic C: official diagnosis states that the diagnosis must be made in conjunction with an ophthalmic examination.

### Characteristic of Incorrect Diagnoses

In the analysis of incorrect top-ranked diagnoses from Hx+Ex profiles, GPT-4.0 exhibited fewer incorrect top-3 diagnoses than GPT-3.5 (25/42, 60% vs 53/63, 84%; *P*=.005), making partially correct diagnoses with incorrect lesion nature (21/42, 50% vs 7/63, 11%; *P*<.001). In contrast, GPT-3.5 often made completely incorrect diagnoses about lesion nature (27/63, 43% vs 7/42, 17%; *P*=.005) and exhibited less precision with no further diagnosis more frequently than GPT-4.0 (22/63, 35% vs 5/42, 12%; *P*=.009; Table 5).

**Table 5:** Analysis of ChatGPT’s incorrect top-ranked diagnoses in patient with history and examination (Hx+Ex) profiles.

			GPT-3.5 (n=63)		GPT-4.0 (n=42)	*P* value	
Proportion of incorrect top-3 diagnosis, n (%)			53 (84)		25 (60)	.005	
Reasons for incorrect top-ranked diagnosis, n (%)							<.001
	Incorrect lesion nature	27 (43)		7 (17)		.005^a^	
	Incorrect etiology	1 (2)		2 (5)		.57	
	Lacks precision, no further diagnosis	22 (35)		5 (12)		.009^a^	
	Partially correct with incorrect lesion location	6 (10)		7 (17)		.28	
	Partially correct with incorrect lesion nature	7 (11)		21 (50)		<.001^a^	

^a^Statistically significant difference between GPT-4.0 and GPT-3.5 at an adjusted *P* value of .01.

## Discussion

### Principal Finding

AI has demonstrated its potential in facilitating accurate patient registration and health care services [13,14]. With iterations, ChatGPT—a chatbot powered by AI—has shown promise for triage in ophthalmic emergencies and in achieving diagnostic accuracy in simulated vignettes [6,15]. Our study focused on investigating the triage and diagnostic value of ChatGPT. To our knowledge, this was the first study to explore the role of ChatGPT in ophthalmic registration. Additionally, we designed 2 types of information inputs, Hx and Hx+Ex profiles, and simultaneously tested the accuracy of leading diagnoses and do-not-miss diagnoses. This approach helped provide a comprehensive understanding of ChatGPT’s performance. While existing studies mainly focus on the application within a single ophthalmic subspecialty [10,11,16,17], another strength of our study was the use of a diverse set of diagnostic cases, covering 12 ophthalmic subspecialties and 104 distinct cases. This breadth enhances the AI system’s evaluation across varied clinical scenarios.

From the patients’ perspective, AI chatbots facilitate ophthalmic triage and appointment [18]. Patients know their own complaints and medical histories well. This knowledge enables them to directly interact with AI chatbots, seeking advice on appropriate ophthalmic subspecialties for registration [4]. Our study used Hx profiles to simulate this self-service interaction, highlighting the practical utility of chatbots in patient-initiated health care navigation. The findings demonstrated that GPT-4.0 directed patients to the correct registration with 78% accuracy, which numerically surpassed the 69% accuracy achieved by medically trained residents. In China, where major tertiary hospitals have implemented web-based registration systems, such as through a WeChat-based medical platform [19], the integration of a user-friendly and accessible AI chatbot significantly streamlines the consultation process for patients, particularly those unsure of which ophthalmic subspecialty to choose. This study provided the first empirical evidence of how AI chatbots can facilitate more accurate and efficient patient registration in clinical settings.

For ophthalmic diagnoses, studies had shown that GPT-4.0 exhibited lower than 50% accuracy in deriving leading diagnoses from complaint records alone [6], while the free version of GPT-3.5 underperforms compared to medical residents [12]. These findings were consistent with our study. In a pilot study by Hu et al [20], GPT-4.0 was tested on its capability to diagnose rare eye diseases, revealing that more comprehensive information provided to the model resulted in considerably more “right” diagnoses. The reduced accuracy when used with limited patient information (such as Hx only) indicated that GPT-4.0 was not yet suitable as a stand-alone diagnostic tool. However, adding details from patient examinations significantly enhanced its performance, making it comparable to that of residents. In real-world clinical settings, where physicians have access to comprehensive case information, GPT-4.0 achieved diagnostic accuracies between 60% to 76%, demonstrating its potential to support clinical decision-making. Similar performance had been observed in uveitis studies, with accuracy rates ranging from 60% to 66% [21,22]. Unlike GPT-3.5, which provided imprecise diagnoses, GPT-4.0, even when erring in its initial diagnosis, tended to include the correct diagnosis within the top 3 suggestions. This illustrated GPT-4.0’s superior information retrieval capabilities and its role as a diagnostic aid [23]. It is expected that with continuous improvements, AI chatbots will play an increasingly vital role in enhancing health care efficiency [24,25].

Our findings revealed that the accuracy of recommendations for ophthalmic subspecialty registration did not consistently correlate with diagnostic accuracy. For example, while the diagnostic accuracy for glaucoma reached 100%, the accuracy of leading patients to register in the glaucoma department was notably low. This discrepancy was likely attributed to the frequently nonspecific initial complaints associated with glaucoma, which generally require additional clinical examinations to establish a definitive diagnosis, such as intraocular pressure, OCT, and visual field tests. Therefore, relying solely on patient complaints and medical histories proved insufficient for accurately guiding patients to the appropriate glaucoma specialty for initial registration. Conversely, when patient histories were supplemented with specific ophthalmic examinations, the accuracy of differential diagnosis improved, thereby enhancing overall diagnostic performance.

Compared to the accuracy of GPT-4.0 in answering ophthalmic questions [26-28] and suggesting surgical plan [29], which can exceed 80%, its performance in registration recommendations and clinical diagnosis was intermediate or inferior. This discrepancy may be attributed to the conservative diagnostic judgment standards and the inclusion of less common diseases. Although the evaluated profiles were derived from real-world clinical reports in the published Chinese SRT database, the inclusion of uncommon and atypical cases was inevitable. Such heightened complexity poses significant challenges not only for AI chatbots but also for residents in the early stages of their medical careers [30]. In real-world clinical settings, the uneven distribution of diseases across various ophthalmic subspecialties typically leads to variability in diagnostic outcomes.

Given that the performance of AI chatbots is contingent upon the volume of information provided, future AI chatbots may still require upgrades and iterations to mitigate information asymmetry between patients and health care professionals, thereby enhancing the delivery of more effective and professional ophthalmic care. For example, AI chatbots specifically designed and trained for ophthalmic care [31,32]; chatbots that can proactively solicit information not provided by end users, similar to the process used by ophthalmologists; and those capable of directly accessing and interpreting imaging data like a multimodal AI chatbot, are essential. Unlike general-purpose AI chatbots such as ChatGPT, Zheng et al [33] have developed a Chinese large language model for ophthalmology using a corpus with extensive clinical vignettes (Hx+Ex). This model demonstrated a diagnostic accuracy of 81.1% across 6 common ophthalmic subspecialties, surpassing the performance of GPT-4.0 (59.6%) in our study. However, the current capability of multimodal GPT-4.0 to diagnose vitreoretinal diseases through the analysis of retinal images remains less than optimal [34].

ChatGPT allows users to customize responses based on personalized prompts, as illustrated in this study by providing 3 differential diagnostic recommendations and the corresponding rationale [35,36]. Research showed that ChatGPT consistently offers a broader range of differential diagnoses than ophthalmology residents [30]; this tendency was also observed in our data collection. The ability to organize key case information through the explanation of diagnostic reasoning not only enhances the knowledge structure of physicians but also underscores the significant educational value of AI chatbots in medical training [37-39]. The proficiency of AI chatbots in responding to ophthalmic examination questions and addressing eye disease queries have been confirmed by recent studies [40-42]. However, it is crucial to note that current AI chatbots do not necessarily replace the clinical judgment of professional ophthalmologists. Their application is still subject to ethical considerations [43,44] and concerns about hallucinations [45]. Physicians responsible for diagnosis need to remain cautious when considering information provided by ChatGPT. The extent to which AI can serve as an adjunct tool in health care still requires further real-world testing.

### Limitations

In clinical settings, ophthalmologists typically rely on direct observation of patients’ symptoms and examinations for intuitive face-to-face diagnoses. However, the text-based format of medical records may not fully reflect ophthalmic residents’ capabilities and might even underestimate them. Additionally, while images are crucial for ophthalmic diagnosis, GPT-4.0’s support for multimodal data still shows suboptimal performance in image-based cases [46]. This explains our decision to exclude images from our evaluation of ChatGPT. However, textual descriptions of these images could have impacted the outcomes. Moreover, although ChatGPT supports multiple languages, differences in language use in diagnosing eye diseases have been observed [11]. Our study, conducted in Mandarin Chinese, may affect the generalization of the results. Despite these limitations, our study contributes to understanding AI’s role as a tool in ophthalmic health care.

### Conclusion

Our study showed that GPT-3.5 and GPT-4.0 demonstrated moderate performance in directing patients to appropriate ophthalmic subspecialties for registration. While GPT-3.5 was less effective, GPT-4.0 approached and even numerically surpassed residents in differential diagnosis when presented with patient histories and examination results. AI chatbots merit emphasis for their potential to facilitate patient registration and optimize consultations in ophthalmology. While their diagnostic capabilities could benefit ophthalmologists, integrating them into diagnostic decision-making still requires further validation.

## Appendix

Multimedia Appendix 1 Prompts for the Hx and Hx+Ex profiles before testing ChatGPT’s registration and diagnosis responses. Hx+Ex: patient with history and examination; Hx: patient with history.

Multimedia Appendix 2 Detailed official subspecialty classification for each clinical profile (Hx), alongside registration recommendations for GPT-3.5, GPT-4.0, and residents. Hx: patient with history.

Multimedia Appendix 3 Detailed official diagnosis and top 3 predicted diagnoses by GPT-3.5 and GPT-4.0, alongside the composite diagnosis by 3 residents (1=correct and 0=incorrect) for each clinical profile (Hx+Ex). Hx+Ex: patient with history and examination.

## Abbreviations

AI: artificial intelligence
Hx: patient with history
Hx+Ex: patient with history and examination
OCT: orbital computed tomography
SRT: Standardized Resident Training

## Data Availability

The raw data supporting the conclusions of this paper will be made available by the authors, without undue reservation.
