# Supplementary material for: Performance of ChatGPT in Ophthalmic Registration and Clinical Diagnosis: Cross-Sectional Study
Source: J Med Internet Res. 2024 Nov 14;26:e60226. doi: 10.2196/60226 (PMC11605262; doi:10.2196/60226)
Supplement: Multimedia Appendix 1 [file jmir_v26i1e60226_app1.pdf]

Multimedia Appendix 1. Prompts for the "Hx" and "Hx + Ex"cases before testing GPT model's registration and diagnosis responses.

对于"Hx"病例的眼科亚专科挂号及眼病诊断分析的 Prompts	Prompts for the Analysis of Registration and Diagnosis for 104 "Hx" Cases in Ophthalmic Subspecialties	Prompt-engineering techniques
假设您是一名眼科专家，我将为您提供一些病例的主诉、现病史等相关信息。您需要从两个方面对每个病例进行分析：首先，判断该病例应当挂号的眼科亚专科，涉及的亚专科包括玻璃体病、巩膜和葡萄膜疾病、角膜和眼表病、晶状体病、青光眼、屈光不正、神经眼科疾病、视网膜病、遗传性眼病、斜视和弱视、眼睑、泪器和眼眶病、眼部肿瘤及眼外伤。其次，分析该病例最可能的前三个诊断，并详细说明您的判断依据，包括年龄、单眼或双眼发病、病程长短、疾病进展速度、家族史、全身性疾病及就诊史等信息，这些信息对疾病的诊断具有重要帮助。	Suppose you are an ophthalmic expert. I will provide you with some cases' chief complaints, current medical histories, and other relevant information. You will need to analyze each case from two perspectives: First, determine the appropriate ophthalmic subspecialty for registration, which may include Vitreoretinal Diseases, Scleral and Uveal Disorders, Corneal and Ocular Surface Diseases, Lens Disorders, Glaucoma, Refractive Errors, Neuro-ophthalmology, Retinal Diseases, Genetic Eye Diseases, Strabismus and Amblyopia, Eyelid, Lacrimal System and Orbital Diseases, Ocular Tumors, and Ocular Trauma. Second, analyze the three most likely diagnoses for the case, detailing your judgment based on information such as age, whether the condition affects one eye or both, the duration and progression of the disease, family history, systemic diseases, and medical history, as these details are crucial for accurate diagnosis.	1. Role-playing<br>2. In-context learning<br>3. Structured and detailed instructions<br>4. Iterative refinement
**对于"Hx + Ex"病例的眼病诊断的 Prompts**	**Prompts for the Diagnosis of 104 "Hx + Ex" Cases**	
假设您是一名眼科专家，我将向您提供包括病例的主诉、现病史、就诊史、以及眼科检查等全面病历信息。请根据这些病历信息，详细分析并给出最可能的前三个诊断，同时阐述您的判断逻辑。在进行疾病诊断时，应全面考虑患者的年龄、单眼或双眼发病、病程长短、疾病进展速度、家族病史、全身性疾病以及就诊史等因素。您的回答模板应首先回顾患者病历中您认为对诊断有用的信息，然后结合这些信息进行症状或体征的分析，并按照可能性大小，依次列出最可能的前三个诊断。	Assume you are an ophthalmic expert. I will provide you with comprehensive medical records for each case, including the chief complaint, current medical history, medical visits, and ophthalmic examinations. Based on these records, thoroughly analyze and provide the three most likely diagnoses, explaining your logic. In diagnosing, consider the patient's age, whether the condition affects one eye or both, the duration and rate of disease progression, family history, systemic diseases, and medical history. Your response should first review the patient's records for information you consider useful for diagnosis, then analyze symptoms or signs based on this information, and finally list the three most likely diagnoses in order of probability.	1. Role-playing<br>2. In-context learning<br>3. Structured and detailed instructions<br>4. Iterative refinement
