# Supplementary material for: Performance of ChatGPT in Ophthalmic Registration and Clinical Diagnosis: Cross-Sectional Study
Source: J Med Internet Res. 2024 Nov 14;26:e60226. doi: 10.2196/60226 (PMC11605262; doi:10.2196/60226)
Supplement: Multimedia Appendix 2 [file jmir_v26i1e60226_app2.docx]

**Multimedia Appendix 2.** Detailed Official Sub-specialty Classification for Each Clinical Case (Hx), Alongside Registration Recommendations for ChatGPT-3.5, GPT-4.0, and Residents.

| **Original**  **Case No.** | **Ophthalmic Sub-specialty** | **Registration suggestion with "Hx"** | | |
| --- | --- | --- | --- | --- |
|  |  | **ChatGPT-3.5** | **GPT-4.0** | **Residents** |
| 7 | Corneal & Ocular Surface Diseases | Eyelid, Lacrimal & Orbital Diseases | Corneal & Ocular Surface Diseases | Corneal & Ocular Surface Diseases |
| 21 |  | Corneal & Ocular Surface Diseases | Scleral & Uveal Diseases | Corneal & Ocular Surface Diseases |
| 25 |  | Eye Tumors | Eyelid, Lacrimal & Orbital Diseases or Corneal & Ocular Surface Diseases | Corneal & Ocular Surface Diseases |
| 30 |  | Eye Tumors | Corneal & Ocular Surface Diseases | Corneal & Ocular Surface Diseases |
| 32 |  | Corneal & Ocular Surface Diseases | Corneal & Ocular Surface Diseases or Lens Diseases | Corneal & Ocular Surface Diseases |
| 48 |  | Corneal & Ocular Surface Diseases | Corneal & Ocular Surface Diseases | Corneal & Ocular Surface Diseases |
| 73 |  | Retinal Diseases or Refractive Errors | Retinal Diseases or Refractive Errors | Retinal Diseases |
| 83 |  | Scleral & Uveal Diseases | Corneal & Ocular Surface Diseases | Corneal & Ocular Surface Diseases |
| 87 |  | Corneal & Ocular Surface Diseases | Corneal & Ocular Surface Diseases | Corneal & Ocular Surface Diseases |
| 2 | Eyelid, Lacrimal & Orbital Diseases | Eyelid, Lacrimal & Orbital Diseases | Eyelid, Lacrimal & Orbital Diseases | Inconsistent answers |
| 4 |  | Eyelid, Lacrimal & Orbital Diseases | Eyelid, Lacrimal & Orbital Diseases | Eyelid, Lacrimal & Orbital Diseases |
| 6 |  | Eyelid, Lacrimal & Orbital Diseases | Strabismus & Amblyopia or Eyelid, Lacrimal & Orbital Diseases | Eyelid, Lacrimal & Orbital Diseases |
| 11 |  | Corneal & Ocular Surface Diseases | Corneal & Ocular Surface Diseases | Corneal & Ocular Surface Diseases |
| 13 |  | Eyelid, Lacrimal & Orbital Diseases | Eyelid, Lacrimal & Orbital Diseases | Eyelid, Lacrimal & Orbital Diseases |
| 19 |  | Eyelid, Lacrimal & Orbital Diseases | Eyelid, Lacrimal & Orbital Diseases | Eyelid, Lacrimal & Orbital Diseases |
| 26 |  | Eyelid, Lacrimal & Orbital Diseases | Eyelid, Lacrimal & Orbital Diseases | Eyelid, Lacrimal & Orbital Diseases |
| 27 |  | Eyelid, Lacrimal & Orbital Diseases | Eyelid, Lacrimal & Orbital Diseases | Corneal & Ocular Surface Diseases |
| 34 |  | Corneal & Ocular Surface Diseases | Corneal & Ocular Surface Diseases | Corneal & Ocular Surface Diseases |
| 43 |  | Eyelid, Lacrimal & Orbital Diseases | Eyelid, Lacrimal & Orbital Diseases | Eyelid, Lacrimal & Orbital Diseases |
| 54 |  | Eyelid, Lacrimal & Orbital Diseases | Eyelid, Lacrimal & Orbital Diseases | Eyelid, Lacrimal & Orbital Diseases |
| 61 |  | Eyelid, Lacrimal & Orbital Diseases | Corneal & Ocular Surface Diseases | Corneal & Ocular Surface Diseases |
| 62 |  | Eyelid, Lacrimal & Orbital Diseases | Eyelid, Lacrimal & Orbital Diseases | Inconsistent answers |
| 72 |  | Eyelid, Lacrimal & Orbital Diseases | Eyelid, Lacrimal & Orbital Diseases | Eyelid, Lacrimal & Orbital Diseases |
| 81 |  | Corneal & Ocular Surface Diseases | Lacrimal Diseases | Eyelid, Lacrimal & Orbital Diseases |
| 84 |  | Eyelid, Lacrimal & Orbital Diseases | Eyelid, Lacrimal & Orbital Diseases | Eyelid, Lacrimal & Orbital Diseases |
| 91 |  | Eyelid, Lacrimal & Orbital Diseases | Eyelid, Lacrimal & Orbital Diseases | Eyelid, Lacrimal & Orbital Diseases |
| 92 |  | Neuro-Ophthalmology | Strabismus & Amblyopia or Neuro-Ophthalmology | Neuro-Ophthalmology |
| 35 | Eyelid, Lacrimal & Orbital Tumors | Scleral & Uveal Diseases | Eyelid, Lacrimal & Orbital Tumors | Eyelid, Lacrimal & Orbital Tumors |
| 37 |  | Eye Tumors | Eyelid, Lacrimal & Orbital Tumors | Eyelid, Lacrimal & Orbital Tumors |
| 65 |  | Eyelid, Lacrimal & Orbital Tumors | Eyelid, Lacrimal & Orbital Tumors | Eyelid, Lacrimal & Orbital Tumors |
| 76 |  | Eyelid, Lacrimal & Orbital Tumors | Eyelid, Lacrimal & Orbital Tumors | Eyelid, Lacrimal & Orbital Tumors |
| 82 |  | Eye Tumors | Eyelid, Lacrimal & Orbital Tumors | Eyelid, Lacrimal & Orbital Tumors |
| 100 |  | Eyelid, Lacrimal & Orbital Tumors | Eyelid, Lacrimal & Orbital Tumors | Eyelid, Lacrimal & Orbital Tumors |
| 9 | Glaucoma | Retinal Diseases | Retinal Diseases | Retinal Diseases |
| 49 |  | Glaucoma | Glaucoma | Glaucoma |
| 55 |  | Heritable Retinal Diseases | Lens Diseases | Inconsistent answers |
| 63 |  | Refractive Errors | Glaucoma or Retinal Diseases | Inconsistent answers |
| 102 |  | Retinal Diseases | Glaucoma or Retinal Diseases | Lens Diseases |
| 14 | Heritable Retinal Diseases | Heritable Retinal Diseases | Heritable Retinal Diseases | Heritable Retinal Diseases |
| 18 |  | Heritable Retinal Diseases | Heritable Retinal Diseases | Heritable Retinal Diseases |
| 22 |  | Heritable Retinal Diseases | Heritable Retinal Diseases | Heritable Retinal Diseases |
| 33 |  | Glaucoma | Corneal & Ocular Surface Diseases | Inconsistent answers |
| 77 |  | Retinal Diseases | Heritable Retinal Diseases | Heritable Retinal Diseases |
| 104 |  | Retinal Diseases | Heritable Retinal Diseases | Heritable Retinal Diseases |
| 28 | Lens Diseases | Lens Diseases | Lens Diseases | Lens Diseases |
| 66 |  | Heritable eye Diseases | Lens Diseases | Inconsistent answers |
| 67 |  | Refractive Errors | Refractive Errors | Refractive Errors |
| 103 |  | Scleral & Uveal Diseases | Scleral & Uveal Tumors | Inconsistent answers |
| 24 | Neuro-Ophthalmology | Neuro-Ophthalmology | Neuro-Ophthalmology | Neuro-Ophthalmology |
| 36 |  | Retinal Diseases | Retinal Diseases or Glaucoma | Retinal Diseases |
| 47 |  | Retinal Diseases | Retinal Diseases | Inconsistent answers |
| 69 |  | Retinal Diseases | Retinal Diseases | Retinal Diseases |
| 1 | Non-heritable Retinal Diseases | Retinal Diseases | Retinal Diseases | Retinal Diseases |
| 10 |  | Retinal Diseases | Retinal Diseases | Retinal Diseases |
| 31 |  | Vitreous Diseases | Non-heritable Retinal Diseases | Lens Diseases |
| 38 |  | Retinal Diseases | Retinal Diseases or Vitreous Diseases | Retinal Diseases |
| 39 |  | Vitreous Diseases | Retinal Diseases or Vitreous Diseases | Retinal Diseases |
| 42 |  | Retinal Diseases | Retinal Diseases | Retinal Diseases |
| 45 |  | Retinal Diseases | Retinal Diseases | Retinal Diseases |
| 50 |  | Retinal Diseases | Retinal Diseases | Retinal Diseases |
| 52 |  | Retinal Diseases | Retinal Diseases | Non-heritable Retinal Diseases |
| 58 |  | Retinal Diseases | Retinal Diseases | Non-heritable Retinal Diseases |
| 64 |  | Retinal Diseases | Retinal Diseases | Retinal Diseases |
| 70 |  | Retinal Diseases | Retinal Diseases | Retinal Diseases |
| 89 |  | Retinal Diseases | Retinal Diseases | Retinal Diseases |
| 90 |  | Retinal Diseases | Retinal Diseases | Retinal Diseases |
| 98 |  | Retinal Diseases | Retinal Diseases | Retinal Diseases |
| 44 | Ocular Trauma | Ocular Trauma | Ocular Trauma | Ocular Trauma |
| 51 |  | Ocular Trauma | Ocular Trauma or Corneal & Ocular Surface Diseases | Ocular Trauma |
| 57 |  | Ocular Trauma | Ocular Trauma | Ocular Trauma |
| 79 |  | Ocular Trauma | Ocular Trauma | Ocular Trauma |
| 96 |  | Ocular Trauma | Ocular Trauma | Ocular Trauma |
| 101 |  | Ocular Trauma | Ocular Trauma | Ocular Trauma |
| 15 | Refractive Errors | Refractive Errors | Strabismus & Amblyopia | Refractive Errors |
| 29 |  | Refractive Errors | Refractive Errors | Refractive Errors |
| 53 |  | Refractive Errors | Refractive Errors or Strabismus & Amblyopia | Refractive Errors |
| 56 |  | Refractive Errors | Refractive Errors or Strabismus & Amblyopia | Refractive Errors |
| 85 |  | Corneal & Ocular Surface Diseases | Corneal & Ocular Surface Diseases | Corneal & Ocular Surface Diseases |
| 93 |  | Refractive Errors | Refractive Errors or Corneal & Ocular Surface Diseases | Inconsistent answers |
| 99 |  | Refractive Errors | Refractive Errors or Corneal & Ocular Surface Diseases | Inconsistent answers |
| 95 | Retinal Tumors | Heritable Retinal Diseases | Retinal Tumors | Retinal Tumors |
| 8 | Scleral & Uveal Diseases | Retinal Diseases | Retinal Diseases or Neuro-Ophthalmology | Inconsistent answers |
| 12 |  | Eyelid, Lacrimal & Orbital Diseases | Scleral & Uveal Diseases | Scleral & Uveal Diseases |
| 23 |  | Retinal Diseases | Vitreous Diseases | Retinal Diseases |
| 40 |  | Eyelid, Lacrimal & Orbital Tumors | Scleral & Uveal Diseases | Scleral & Uveal Diseases |
| 59 |  | Scleral & Uveal Diseases | Scleral & Uveal Diseases | Scleral & Uveal Diseases |
| 78 |  | Corneal & Ocular Surface Diseases | Scleral & Uveal Diseases or Corneal & Ocular Surface Diseases | Inconsistent answers |
| 3 | Scleral & Uveal Tumors | Retinal Diseases | Eye Tumors | Eye Tumors |
| 20 |  | Eye Tumors | Retinal Diseases or Neuro-Ophthalmology | Eye Tumors |
| 75 |  | Retinal Diseases | Retinal Diseases or Neuro-Ophthalmology | Retinal Diseases |
| 88 |  | Refractive Errors | Retinal Diseases or Refractive Errors | Strabismus & Amblyopia |
| 5 | Strabismus & Amblyopia | Strabismus & Amblyopia | Strabismus & Amblyopia | Strabismus & Amblyopia |
| 16 |  | Strabismus & Amblyopia | Strabismus & Amblyopia | Strabismus & Amblyopia |
| 41 |  | Strabismus & Amblyopia | Strabismus & Amblyopia | Strabismus & Amblyopia |
| 46 |  | Strabismus & Amblyopia or Neuro-Ophthalmology | Strabismus & Amblyopia or Neuro-Ophthalmology | Neuro-Ophthalmology |
| 68 |  | Strabismus & Amblyopia | Strabismus & Amblyopia or Neuro-Ophthalmology | Strabismus & Amblyopia |
| 71 |  | Strabismus & Amblyopia | Eyelid, Lacrimal & Orbital Diseases | Eyelid, Lacrimal & Orbital Diseases |
| 74 |  | Neuro-Ophthalmology | Strabismus & Amblyopia or Neuro-Ophthalmology | Neuro-Ophthalmology |
| 80 |  | Strabismus & Amblyopia | Strabismus & Amblyopia | Strabismus & Amblyopia |
| 86 |  | Strabismus & Amblyopia | Strabismus & Amblyopia | Strabismus & Amblyopia |
| 97 |  | Refractive Errors | Strabismus & Amblyopia | Strabismus & Amblyopia |
| 17 | Vitreous Diseases | Retinal Diseases | Vitreous Diseases | Vitreous Diseases |
| 60 |  | Retinal Diseases | Vitreous Diseases | Vitreous Diseases |
| 94 |  | Heritable Retinal Diseases | Eye Tumors | Eye Tumors |
