# Supplementary material for: Performance of ChatGPT in Ophthalmic Registration and Clinical Diagnosis: Cross-Sectional Study
Source: J Med Internet Res. 2024 Nov 14;26:e60226. doi: 10.2196/60226 (PMC11605262; doi:10.2196/60226)
Supplement: Multimedia Appendix 3 [file jmir_v26i1e60226_app3.docx]

**Multimedia Appendix 3.** Detailed Official Diagnosis and Top-3 Predicted Diagnoses by ChatGPT-3.5 and GPT-4.0, Alongside the Composite Diagnosis by Three Residents (1= Correct, 0 = Incorrect) for Each Clinical Case (History + Examination).

| **Case No.** | **Official diagnosis** | **Top three diagnoses of ChatGPT-3.5** | | | **Top three diagnoses of GPT-4.0** | | | **Residents** | |
| --- | --- | --- | --- | --- | --- | --- | --- | --- | --- |
|  |  | **Diagnosis 1** | **Diagnosis 2** | **Diagnosis 3** | **Diagnosis 1** | **Diagnosis 2** | **Diagnosis 3** | **Top - diagnosis** | **Top-3 diagnosis** |
| 1 | Retinal artery occlusion, RAO | Retinal artery occlusion | Vitreous hemorrhage | Premacular membrane | Central Retinal Artery Occlusion | Retinal Vein Occlusion | Transient Ischemic Attack | **1** | **1** |
| 2 | Thyroid eye disease | Thyroid eye disease | Orbital tumor | Glaucoma | Thyroid eye disease | Orbital inflammation or infection | Orbital tumor | 0 | 0 |
| 3 | Circumscribed choroidal hemangioma | Choroidal melanoma | Retinal detachment | Macular disease | Choroidal melanoma | Retinal pigment epithelioma | Choroidal metastasis | 1 | 1 |
| 4 | Chronic dacryocystitis | Lacrimal duct obstruction | Dacryocystitis | Keratitis | Dacryocystitis (acute or chronic) | Lacrimal duct obstruction | Chronic conjunctivitis | 1 | 1 |
| 5 | Concomitant strabismus | Strabismus | Eye muscle paralysis | Amblyopia | Concomitant strabismus | Basic exotropia | Congenital exotropia | 1 | 1 |
| 6 | Congenital ptosis | Congenital upper eyelid ptosis | Horner's syndrome | Marfan syndrome | Congenital eyelid ptosis | Horner's syndrome | Third cranial nerve palsy | 1 | 1 |
| 7 | Viral conjunctivitis | Acute viral conjunctivitis | Conjunctival tuberculosis | NA | Viral conjunctivitis | Bacterial conjunctivitis | Conjunctivitis - lymphatic syndrome (e.g., adenovirus) | 1 | 1 |
| 8 | Behçet's disease | Retinal pigmentary changes | Secondary cataract | Systemic disease impact | Behçet's disease | Uveitis | Systemic Lupus Erythematosus | 1 | 1 |
| 9 | Secondary glaucoma | Neovascular glaucoma | Diabetic retinopathy | Lens opacification | Neovascular glaucoma | Post-diabetic retinopathy sequelae | Complications post-photocoagulation | 1 | 1 |
| 10 | Vitreomacular traction syndrome | Macular edema | Vitreomacular adhesion | Macular degeneration | Macular hole | Premacular membrane | Macular edema | 0 | 1 |
| 11 | Entropion | Entropion | Conjunctivitis | NA | Entropion | Trichiasis | Chronic conjunctivitis | 0 | 0 |
| 12 | Vogt-Koyanagi-Harada syndrome, VKH | Retinal detachment | Iritis ciliary body | Vitreous opacity | Vogt-Koyanagi-Harada syndrome | Systemic autoimmune disease | Viral uveitis | 1 | 1 |
| 13 | Orbital cellulitis | Orbital abscess | Eye movement disorder | Orbital inflammation | Orbital abscess | Periorbital cellulitis | Infectious orbital cellulitis | 1 | 1 |
| 14 | Leber's congenital amaurosis, LCA | Hereditary retinal disease | Hereditary nystagmus | Hereditary cataract | Retinitis Pigmentosa | Congenital Vitreoretinopathy | Leber Congenital Amaurosis | 0 | 0 |
| 15 | Myopia | Myopia | Amblyopia | Eye strain | Refractive error (myopia) | Latent strabismus | Accommodative exotropia | 1 | 1 |
| 16 | Infantile esotropia | Isolated strabismus | Congenital strabismus | Sensory strabismus | Congenital Esotropia | Refractive strabismus | Accommodative esotropia | 1 | 1 |
| 17 | Posterior vitreous detachment, PVD | Posterior vitreous detachment | Retinal tear | Vitreous hemorrhage | Posterior Vitreous Detachment | Peripheral retinal tear | Retinal tear | 0 | 1 |
| 18 | Stargardt disease | Stargardt's disease | Age-related macular degeneration | Other hereditary macular diseases | Stargardt's disease | Age-related macular degeneration | Hereditary macular degeneration | 1 | 1 |
| 19 | Lacrimal gland pleomorphic adenoma | Orbital tumor | Proptosis | Thyroid eye disease | Lacrimal gland pleomorphic adenoma | Orbital hemangioma | Osseous orbital tumor | 0 | 0 |
| 20 | Choroidal metastatic cancer | Choroidal metastatic carcinoma | Breast cancer lung metastasis | NA | Choroidal metastatic tumor | Breast cancer ocular metastasis | Breast cancer bilateral lung and lymph node metastasis | 1 | 1 |
| 21 | Bullous keratopathy | Keratitis | Corneal ulcer | Corneal neovascularization | Keratitis | Chronic surface eye disease | Corneal neovascularization | 0 | 0 |
| 22 | Retinitis pigmentosa, RP | Retinitis pigmentosa | Optic nerve disease | Hereditary fundus disease | Retinitis pigmentosa | Retinal detachment or other retinal diseases | Cone rod dystrophy | 1 | 1 |
| 23 | Intermediate uveitis | Vitreous haze | Peripheral retinal vasculitis | Other retinal diseases | Uveitis | Peripheral retinal disease | Vitreous disease | 1 | 1 |
| 24 | Acute idiopathic optic neuritis, TON | Optic neuritis | Visual field defect | Multiple sclerosis | Acute idiopathic optic neuritis | MS-related optic nerve disease | Demyelinating disease of the central nervous system | 1 | 1 |
| 25 | Pterygium | Corneal Granuloma | Cataract | NA | Pterygium | Conjunctival hyperplasia or scarring | Chronic conjunctivitis | 1 | 1 |
| 26 | Acquired nasolacrimal duct obstruction | Acquired lacrimal duct obstruction | NA | NA | Lacrimal duct obstruction | Dacryocystitis | Dry eye syndrome | 1 | 1 |
| 27 | Herpes Zoster Ophthalmicus | Viral conjunctivitis | Allergic conjunctivitis | Bacterial conjunctivitis | Herpes zoster ophthalmicus | Preseptal bacterial conjunctivitis or blepharitis | Viral conjunctivitis | 1 | 1 |
| 28 | Age-related cataract | Cataract | Glaucoma | Macular degeneration | Age-related cataract | Dry (non-exudative) age-related macular degeneration | Opacification of the refractive media | 1 | 1 |
| 29 | Accommodative insufficiency | Refractive error | Strabismus | Other eye diseases | Refractive error | Accommodative insufficiency | Binocular vision dysfunction | 0 | 0 |
| 30 | Corneal dermoid tumor | Eyelid lipoma | Other eyelid lesions | NA | Corneal dermoid tumor | Lipoma | Conjunctival cyst | 0 | 0 |
| 31 | Coats disease | Hereditary retinal pigmentary changes | Retinal vascular diseases | Other retinal diseases | Retinoblastoma | Coats disease | Congenital retinal vascular malformation | 1 | 1 |
| 32 | Bullous keratopathy | Corneal edema | Complications related to artificial intraocular lenses | Other anterior segment diseases | Corneal endothelial cell dysfunction | Secondary corneal disease | Chronic anterior uveitis | 1 | 1 |
| 33 | Cone dystrophy | Retinitis Pigmentosa | Other retinal diseases | NA | Cone retinopathy | Macular degeneration | Hereditary retinal disease | 0 | 0 |
| 34 | Seborrheic blepharitis | Eyelid dermatitis | Conjunctivitis | Dry eye syndrome | Seborrheic blepharitis | Seborrheic or dandruff-related eye disease | Allergic conjunctivitis | 1 | 1 |
| 35 | Orbital schwannoma | Orbital tumor | Thyroid eye disease | Other orbital diseases | Orbital tumor | Mucocele or cyst | Graves' ophthalmopathy | 1 | 1 |
| 36 | Tobacco-alcohol optic neuropathy | Age-related macular degeneration | Glaucoma | Other retinal diseases | Early optic atrophy | Toxic (tobacco-alcohol) optic neuropathy | Ischemic optic neuropathy | 0 | 0 |
| 37 | Eyelid hemangioma | Eyelid vascular lesion | Eyelid dermatological condition | Eyelid inflammatory condition | Infantile eyelid hemangioma | Capillary malformation | Skin inflammatory reaction | 1 | 1 |
| 38 | Rhegmatogenous retinal detachment, RRD | Rhegmatogenous retinal detachment | Posterior vitreous detachment | Retinal tear | Rhegmatogenous Retinal detachment | Retinal vascular blockage | Retinal or vitreous hemorrhage | 1 | 1 |
| 39 | Eales disease | Vitreous hemorrhage | Retinal detachment | Diabetic retinopathy | Vitreous hemorrhage | Retinal vascular anomalies | Systemic disease-related eye complications | 0 | 0 |
| 40 | Scleritis | Rheumatoid arthritis-induced scleritis | NA | NA | Scleritis | Anterior uveitis | Dry eye syndrome | 1 | 1 |
| 41 | Congenital oculomotor nerve palsy | Congenital exotropia with weakness | Congenital strabismus | Congenital exotropia | Congenital eye muscle paralysis | Duane syndrome | Congenital eyelid ptosis | 1 | 1 |
| 42 | Central retinal vein occlusion, CRVO | Central retinal vein occlusion | Macular edema | NA | Retinal vein occlusion | Proliferative diabetic retinopathy | Macular degeneration | 0 | 0 |
| 43 | Meibomian gland cyst | Meibomian cyst | Meibomian gland cyst | Palpebral conjunctival cyst | Meibomian gland cyst | Meibomian gland adenoma | Epidermal cyst | 0 | 0 |
| 44 | Intravitreal foreign bodies | Foreign bodies into eye | Corneal abrasion or damage | Conjunctivitis | Foreign body injury | Corneal abrasion | Conjunctivitis | 0 | 0 |
| 45 | Idiopathic macular hole | Macular hole | Macular disease | Retinal edema | Macular pucker | Macular hole | Early stage of retinal detachment | 0 | 0 |
| 46 | Acquired paralytic strabismus | Right abducens nerve palsy | Lateral rectus paralysis | Third cranial nerve palsy | Abducens nerve palsy | Eye muscle disorder or dysfunction | Intracranial disease | 0 | 0 |
| 47 | Compressive optic neuropathy | Pituitary growth hormone adenoma | Optic nerve damage | Pituitary disease (other types of pituitary tumors) | Pituitary growth hormone-induced optic nerve compression | Intracranial tumor-induced optic nerve disease | Optic neuropathy | 0 | 1 |
| 48 | Dry eye syndrome | Dry eye syndrome | Conjunctivitis | Possible corneal disease | Dry eye syndrome | Meibomian gland dysfunction | Chronic conjunctivitis | 1 | 1 |
| 49 | Acute angle-closure glaucoma | Acute angle-closure glaucoma attack | Possible optic nerve compression | Meningitis-related eye disease | Acute angle-closure glaucoma | Subacute angle-closure glaucoma | Acute anterior uveitis | 1 | 1 |
| 50 | Diabetic retinopathy | Diabetic retinopathy | Macular disease | Retinal vein occlusion | Diabetic retinopathy | Macular edema | Non-proliferative diabetic retinopathy | 1 | 1 |
| 51 | Ocular chemical burn | Chemical corneal burn | NA | NA | Chemical corneal burn | Acute conjunctivitis | Keratitis | 1 | 1 |
| 52 | Pathologic myopia | Pathologic myopia | Retinal Pigment Epithelial Detachment | Diabetic retinopathy | Pathologic myopia | Myopic choroidal neovascularization | Macular retinal detachment | 0 | 0 |
| 53 | Astigmatism | Myopia | Corneal astigmatism | NA | Refractive error | Astigmatism discomfort | Irregular astigmatism | 0 | 0 |
| 54 | Dacryoadenitis | Eyelid fat tumor | Orbital mass | Other causes of eyelid edema | Eyelid cyst or lipoma | Chronic eyelid inflammation | Eyelid fat accumulation (eye bags) | 0 | 0 |
| 55 | Primary congenital glaucoma | Congenital glaucoma | Congenital cataract | Ocular developmental anomalies | Congenital glaucoma | Corneal disease | Developmental ocular disease | 1 | 1 |
| 56 | Anisometropia | Refractive error | Amblyopia | Strabismus | Refractive error | Amblyopia | Strabismus | 0 | 0 |
| 57 | Ciliary body detachment | Ocular contusion | Hyphema | Ciliary body injury | Ciliary body detachment | Hyphema | Post-traumatic retinal disease | 0 | 1 |
| 58 | Macular epiretinal membrane | Macular degeneration | Retinal tear | Retinal detachment | Dry age-related macular degeneration | Wet age-related macular degeneration | Macular hole | 0 | 0 |
| 59 | Sympathetic ophthalmia | Uveitis | Complications post-corneal perforation | Retinal edema | Uveitis | Secondary glaucoma | Posterior segment eye disease | 0 | 0 |
| 60 | Asteroid hyalosis | Vitreous haze | Vitreous laser fragments | Vitreous hemorrhage | Vitreous floaters | Posterior vitreous detachment | Vitreous hemorrhage | 0 | 0 |
| 61 | Sjögren's syndrome | Sjögren's syndrome | NA | NA | Sjögren's syndrome | Eye dryness not caused by Sjögren's | Other autoimmune diseases | 1 | 1 |
| 62 | Graves' ophthalmopathy | Graves' eye disease | NA | NA | Graves' ophthalmopathy | Extraocular muscle disorder | Optic nerve compression | 1 | 1 |
| 63 | Primary open-angle glaucoma, POAG | Primary open-angle glaucoma | NA | NA | Primary open-angle glaucoma | Optic nerve disease | Retinal nerve fiber layer defect | 1 | 1 |
| 64 | Vitreous hemorrhage | Acute retinal hemorrhage | Retinal detachment | Retinal artery occlusion | Vitreous hemorrhage | Proliferative diabetic retinopathy | Retinal vein occlusion | 1 | 1 |
| 65 | Orbital cavernous hemangioma | Orbital tumor | Myositis ossificans | Other orbital tumors | Orbital tumor | Optic nerve glioma | Orbital schwannoma | 1 | 1 |
| 66 | Congenital cataract | Congenital cataract | Retinoblastoma | Vitreous hemorrhage | Congenital cataract | Hereditary eye disease | Metabolic disease | 1 | 1 |
| 67 | Lens subluxation and dislocation | High myopia | Significant increase in astigmatism | Corneal deformation | Increased corneal astigmatism | Lens abnormality or dislocation | Progression of refractive error | 1 | 1 |
| 68 | Duane's retraction syndrome, DRS | Paralysis of extraocular muscle | Dysfunction of extraocular muscle | Abnormal exotropia with extraocular muscle anomaly | Left abducens nerve palsy | Congenital strabismus | Dysfunction of the lateral rectus muscle | 0 | 0 |
| 69 | Non-arteritic anterior ischemic optic neuropathy, NAION | Hypertensive retinopathy | Retinal artery occlusion | Choroidal ischemic retinopathy | Optic neuropathy | Non-arteritic anterior ischemic optic neuropathy | Hypertensive retinopathy | 0 | 0 |
| 70 | Branch retinal vein occlusion, BRVO | Retinal vein occlusion | Macular edema | Hypertensive fundus disease | Retinal vein occlusion | Hypertensive retinopathy | Age-related macular degeneration | 0 | 0 |
| 71 | Congenital fibrosis of extraocular muscles, CFEOM | Congenital strabismus | Congenital dysfunction of extraocular muscles | Congenital muscular strabismus | Congenital eyelid ptosis | Extraocular muscle disease or paralysis | Comitant esotropia | 0 | 0 |
| 72 | Meibomitis | Blepharitis | Infected meibomian cyst | Eyelid abscess | Eyelid cellulitis | Bacterial skin infection | Infectious eyelid ulcer | 1 | 1 |
| 73 | Corneal Dystrophy | Corneal opacity | Diffuse corneal malnutrition | Corneal leukoma | Corneal opacification | Corneal dystrophy | Corneal inflammation or disease | 0 | 0 |
| 74 | Thyroid Eye Disease, TED | Paralysis of cranial nerves III, IV, VI | Paralytic strabismus | Thyroid eye disease | Thyroid eye disease | Extraocular muscle paralysis or dysfunction | Strabismus | 1 | 1 |
| 75 | Choroidal melanoma | Choroidal melanoma | Choroidal detachment | Non-secondary retinal detachment | Choroidal melanoma | Choroidal metastatic tumor | Choroidal vascular disease | 0 | 0 |
| 76 | Eyelid melanocytic nevus | Eyelid melanocytic nevus | Papilloma | Lipoma | Eyelid melanocytic nevus | Melanoma | Pigmented skin proliferation | 1 | 1 |
| 77 | Best disease | Age-related macular degeneration | Stargardt's disease | Macular hole | Patter dystrophy | Best's vitelliform macular dystrophy | Early-onset age-related macular degeneration | 0 | 0 |
| 78 | Anterior scleritis | Eyelid inflammation | Anterior chamber inflammation | Vitreous haze | Anterior scleritis | Corneal inflammation | Intraocular inflammation | 1 | 1 |
| 79 | Eyelid laceration involving lacrimal apparatus | Left eyelid laceration | Left eyelid split | Eyelid tissue damage and bleeding | Traumatic eyelid laceration | Conjunctival hemorrhage | Possible eyelid structural damage | 1 | 1 |
| 80 | Dissociated vertical deviation, DVD | Congenital strabismus | Congenital ocular motor disorder | Congenital abnormal ocular positioning | Congenital extraocular muscle paralysis | Optic nerve or brain issues | Comitant strabismus | 1 | 1 |
| 81 | Ectropion | Lacrimal tract dysfunction | Dry eye syndrome | Eyelid dysfunction | Dry eye syndrome | Ectropion | Chronic conjunctivitis | 1 | 1 |
| 82 | Eyelid malignancy | Skin melanoma | Skin malignant melanoma | Benign tumor malignancy | Melanoma | Eyelid basal cell carcinoma | Squamous cell carcinoma | 1 | 1 |
| 83 | Fungal keratitis | Bacterial corneal ulcer | Fungal keratitis | Anterior chamber pus | Corneal ulcer | Bacterial keratitis | Fungal keratitis | 1 | 1 |
| 84 | Congenital nasolacrimal duct obstruction | Lacrimal duct obstruction | Conjunctivitis | Abnormal development of the lacrimal duct | Congenital nasolacrimal duct obstruction | Conjunctivitis | Chronic dacryocystitis | 1 | 1 |
| 85 | Presbyopia | Eye strain | Dry eye syndrome | Insufficient myopia correction | Eye strain | Dry eye syndrome | Refractive error | 0 | 0 |
| 86 | High myopic esotropia | High myopic esotropia | Latent strabismus | Ocular motor disorder | High myopic strabismus | Eye muscle dysfunction | Eye muscle paralysis or palsy | 1 | 0 |
| 87 | Immune conjunctivitis (Keratoconjunctivitis) | Allergic conjunctivitis | Allergic keratitis | Seasonal allergic conjunctivitis | Allergic conjunctivitis | Chronic conjunctivitis | Dry eye symptoms | 1 | 1 |
| 88 | Choroidal osteoma | Macular degeneration | Central serous choroidoretinopathy | Choroiditis | Choroidal tumor | Retinal-choroidal detachment | Central serous chorioretinopathy | 1 | 1 |
| 89 | Age-related macular degeneration, AMD | Age-related macular degeneration | Central retinal vein occlusion | Secondary to macular degeneration macular hole | Age-related macular degeneration | Macular hole | Macular edema | 1 | 1 |
| 90 | Central serous chorioretinopathy, CS | Central retinal pigment epithelium disease | Optic nerve disease | Nonspecific macular disease | Central serous chorioretinopathy | Macular disease | Retinal detachment | 0 | 0 |
| 91 | Ptosis | Ptosis | Congenital ptosis | NA | Ptosis | Neurogenic eyelid ptosis | Myogenic eyelid ptosis | 0 | 1 |
| 92 | Orbital nonspecific myositis | Nonspecific orbital myositis | Extraocular muscle inflammation | Fundus disease | Idiopathic orbital myositis | Thyroid eye disease | Cranial nerve-induced eye muscle paralysis | 0 | 0 |
| 93 | Insufficient accommodation | Eye fatigue | Refractive error | NA | Eye strain | Refractive error | Insufficient accommodation or eye muscle coordination | 0 | 0 |
| 94 | Persistent hyperplastic primary vitreous, PHPV | Congenital cataract | Abnormal ocular development | Abnormal vitreous attachment | Persistent hyperplastic primary vitreous | Congenital cataract | Retinoblastoma | 0 | 0 |
| 95 | Retinoblastoma, RB | Fundus tumor | Diffuse vitreous hyperplasia | Retinal disease | Retinoblastoma | Congenital cataract | Persistent hyperplastic primary vitreous | 1 | 1 |
| 96 | Traumatic corneoscleral laceration | Traumatic corneoscleral laceration | Ocular injury | Orbital fracture | Traumatic corneoscleral laceration | Iris incarceration | Lens injury or anterior chamber hemorrhage | 1 | 1 |
| 97 | Amblyopia | Amblyopia | Refractive error | Retinal or optic nerve disease | Refractive error | Potential intraocular disease | Amblyopia | 1 | 1 |
| 98 | Epiretinal Membrane | Macular degeneration | Macular edema | Retinal detachment | Age-related macular degeneration | Epiretinal membrane | Central serous chorioretinopathy | 1 | 1 |
| 99 | Hyperopia | Refractive error | Phoria | Ocular muscle paralysis | Hyperopia | Astigmatism | Accommodative esotropia | 0 | 0 |
| 100 | Orbital neurofibroma | Orbital tumor | Neurofibroma | Other orbital mass | Eyelid lymphangioma | Localized fibrous tumor | Orbital tumor | 1 | 1 |
| 101 | Traumatic optic neuropathy | Dilated retinopathy | Pupil motility abnormality | Traumatic optic nerve injury | Oculomotor nerve palsy | Mild traumatic brain injury | Minor ocular trauma | 1 | 1 |
| 102 | Chronic angle-closure glaucoma | Angle-closure glaucoma | Cataract | Optic nerve disease | Primary angle-closure glaucoma | Optic nerve disease | Cataract | 1 | 1 |
| 103 | Complicated cataract | Iridocyclitis | Chronic intraocular inflammation | Lens opacification | Secondary cataract | Recurrent iridocyclitis | Rheumatic eye disease | 0 | 1 |
| 104 | Familial exudative vitreoretinopathy, FEVR | Familial Exudative Vitreoretinopathy | Hereditary strabismus | Retinal detachment | Familial Exudative Vitreoretinopathy | Hereditary retinal detachment | Congenital retinal dysplasia | 1 | 1 |
